# Supplementary material for: Content-rich biological network constructed by mining PubMed abstracts
Source: BMC Bioinformatics. 2004 Oct 8;5:147. doi: 10.1186/1471-2105-5-147 (PMC528731; doi:10.1186/1471-2105-5-147)
Supplement: Additional File 2 — The original results of the above study (non-essential files are deleted to keep the file size under the limit set by BMC bioinformatics). [file 1471-2105-5-147-S2.bz2 › chilibotAdditionalFile2/dip05/159ID7685215E561/html/JUN_TBP.html]

 


 **JUN** and **TBP** 
  
Found 81 abstracts in PubMed, retrieved 05.  
 

 What does Google say? 
 PDF only 
| .edu only 

---

**Interactive relationship** (e.g. stimulation, inhibition, etc)

**Non-interactive relationship** (e.g. studied together, co-existance, homology, etc.)

- Strikingly, the first modification observed is methylation of histone H3 lysine 4, which correlates with the binding of the SET9 methyltransferase and the assembly of a complex consisting of c  **Jun** , c Fos, TATA binding protein  [ **TBP** ] , and RNA polymerase II.  Ref: 12588998 Mol Cell Biol, 2003
- Scanning the BPV 1 genome for potential binding sequences, an intragenic AP 1  [ **JUN** ]  site BAP  [ **TBP** ]  1 within the E7 open reading frame was detected.  Ref: 12867421 J Biol ChemJ Biol Chem, 2003
- Chromatin immunoprecipitation analyses demonstrated that T cell activation triggers in vivo binding of endogenous c Rel, c  **Jun** , C EBP beta, and HMG IC to the bfl 1 regulatory region, coincident with selective recruitment of coactivators TAFII250 and p300, SWI SNF chromatin remodeling factor component BRG 1, and basal transcription factors TATA binding protein  [ **TBP** ]   **TBP**  and TFIIB, as well as hyperacetylation of histones H3 and H4.  Ref: 12665576 Mol Cell Biol, 2003
- In the present study we have analysed the mechanism of c  **Jun**  function as a trans activator with respect to members of the basal transcription machinery, TATA binding protein  [ **TBP** ]  associated factors TAFs.  Ref: 12676957 J Biol Chem< MedlineTA>J Biol Chem, 2003
- Enhanced dimerization of phosphorylated ATF 2 together with c  **Jun**  and binding to BAP  [ **TBP** ]  1 seems to be responsible for viral dysregulation, since both suppression of BPV 1 and c  **jun**  mRNA induction could be almost entirely abrogated by simultaneous treating with SB 203580, an inhibitor of p38 MAP kinase activity.  Ref: 12867421 J Biol ChemJ Biol Chem, 2003
